# All-trans retinoic acid enhances cytotoxic effect of T cells with an anti-CD38 chimeric antigen receptor in acute myeloid leukemia

**DOI:** 10.1038/cti.2016.73

**Published:** 2016-12-09

**Authors:** Tetsumi Yoshida, Keichiro Mihara, Yoshifumi Takei, Kazuyoshi Yanagihara, Takanori Kubo, Joyeeta Bhattacharyya, Chihaya Imai, Tatsuji Mino, Yoshihiro Takihara, Tatsuo Ichinohe

**Affiliations:** 1 Department of Hematology and Oncology, Research Institute for Radiation Biology and Medicine, Hiroshima University, Hiroshima, Japan; 2 Department of Biochemistry, Nagoya University Graduate School of Medicine, Nagoya, Japan; 3 Division of Translational Research, Exploratory Oncology Research and Clinical Trial Center, National Cancer Center, Chiba, Japan; 4 Department of Life Sciences, Yasuda Women's University Faculty of Pharmacy, Hiroshima, Japan; 5 Department of Pediatrics, Niigata University School of Medicine, Niigata, Japan; 6 Department of Stem Cell Biology, Research Institute for Radiation Biology and Medicine, Hiroshima University, Hiroshima, Japan

## Abstract

We reported that T cells with anti-CD38-chimeric antigen receptors (CAR) eliminated B-cell lymphoma cells expressing CD38. To employ anti-CD38-CAR against acute myeloid leukemia (AML) blasts not expressing CD38, it is necessary to induce or increase the intensity of CD38 expression. A lactate dehydrogenase (LDH)-releasing assay and flow cytometry showed that anti-CD38-CAR T cells were cytotoxic against AML lines (THP-1 and CMK) expressing high CD38 levels (>99%), in time- and number of effector-dependent manners. In other AML lines (KG1, U937 and HL60) partially expressing CD38, CD38^+^ AML cells were killed by CD38-specific T cells, but CD38^−^ AML cells remained survived. Intriguingly, 10 nM all-trans retinoic acid (ATRA) augmented CD38 expression in KG1, U937 and HL60 cells and primary leukemic cells from AML patients. Moreover, the withdrawal of ATRA from the medium decreased CD38 expression in AML cells. Killing effects of anti-CD38-CAR T cells against AML lines and AML cells were limited without ATRA, whereas CD38-specific T cells enhanced cytotoxicity on AML cells by ATRA in association with enhanced CD38 expression. These results indicate that anti-CD38-CAR T cells eliminate AML cells through CD38 expression induced by ATRA.

Acute myeloid leukemia (AML) is a heterogeneous group of clonal hematopoietic neoplasms that increasingly occur in the elderly population. Conventional chemotherapy and hematopoietic stem cell (HSC) transplantation, albeit with substantial toxicities, can cure 20–75% of younger or fit patients with AML depending on the subtypes and genetic properties of leukemia.^1, 2^ However, long-term survival can be expected in less than 10% of elderly or debilitated patients with AML because they frequently cannot tolerate dose-intensive or toxic treatment.^1, 2^ The prognosis of patients with primary resistant or relapsed AML is also poor, although a small proportion of them can be rescued by allogeneic HSC transplantation. Therefore, to improve the outcomes of these subgroups of poor-risk AML patients, the development of a more effective molecular-targeted therapeutic strategy with less adverse effects has been strongly warranted for a long period of time.

To date, T cells transduced with a genetic modified chimeric antigen receptor (CAR) to CD19 have had a clinically marked impact on patients with B-cell chronic lymphocytic leukemia and B-cell acute lymphoblastic leukemia, which are highly refractory and relapsed.^3, 4, 5, 6, 7, 8^ Patients injected with T cells harboring anti-CD19-CAR through the peripheral blood achieved complete and sustained remission, although T cells with anti-CD19-CAR unfortunately caused prolonged B-cell aplasia in these patients. Thus, an adoptive immunotherapy with T cells bearing CAR is expected to be a promising tool for refractory hematological disorders.^9^ To apply this strategy for patients with AML, it is necessary to identify another suitable molecular target expressed on the surface of AML blasts that do not usually express CD19.

Although human HSCs share CD34^+^ without CD38, the majority of AML blasts express CD38.^10, 11^ Accordingly, we focused on CD38 as a candidate therapeutic target and developed anti-CD38-CAR. We recently reported that T cells with anti-CD38-CAR efficiently eliminated B-cell lymphoma cells and myeloma cells expressing CD38 *in vitro* and *in vivo*.^12, 13, 14, 15, 16^ However, the intensity and number of CD38 in lymphoma or myeloma cells are much higher than in AML cells. Thus, to fully employ anti-CD38-CAR against AML blasts, the intensity of CD38 expression must be raised for clinical application.

All-trans retinoic acid (ATRA) is widely used for the treatment of acute promyelocytic leukemia (APL). This compound can induce the differentiation of APL cells, leading to effective suppression of the proliferation capacity of these cells. Alternatively, the use of the reagent has a low risk of critical adverse effects, including acute pulmonary edema, which is known as an ATRA syndrome. On the other hand, it has been reported that it can enhance the expression of CD38 on the surface of AML cells such as HL60.^17, 18, 19, 20^

In this study, we exploited an inducible immunotherapeutic option to enhance CD38 expression of AML cells using ATRA for the application of T cells with anti-CD38-CAR *in vitro*, and suggest their useful combination in a new therapeutic strategy for AML.

## Results

### Cytotoxic effect of T cells with anti-CD38-CAR against AML cells

**Figure 1:**
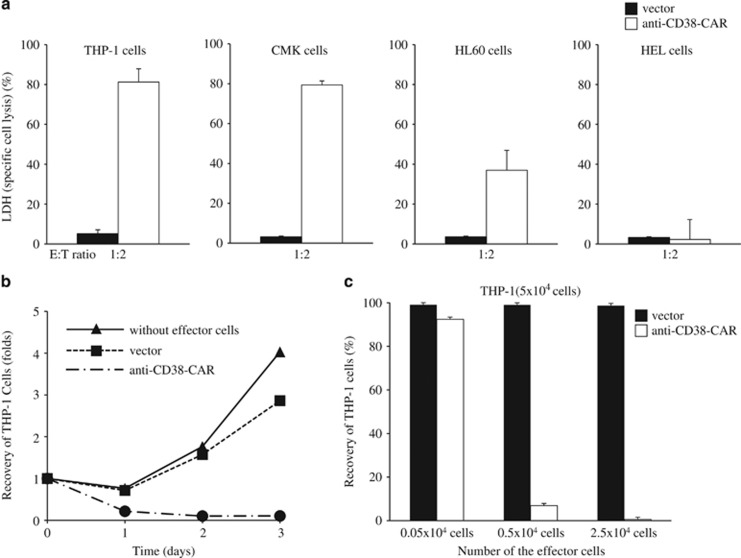
Cytotoxicity of T cells with anti-CD38-CAR against AML cell lines. (**a**) Three AML cell lines, THP-1 cells highly expressing CD38 (>99% CD38 expression rate), HL60 cells partially expressing CD38 (35.05%) and HEL cells without CD38 expression, were co-cultured with T cells transduced with an empty vector or anti-CD38-CAR at an E:T ratio of 1:2 for 3 days. Cytotoxicity (specific cell lysis) was evaluated with the LDH-releasing assay. (**b**) THP-1 cells were co-incubated with T cells transduced at an E:T ratio of 1:2 for 3 consecutive days *in vitro*. The cells recovered from the culture vessel were stained with an anti-CD38 antibody conjugated to APC. Stained cells were analyzed with a flow cytometer for evaluation of the cytotoxicity of T cells bearing anti-CD38-CAR. (**c**) THP-1 cells were co-cultured with T cells transduced at a variety of E:T ratios for 3 days. Cells stained with anti-CD38 antibody-APC were subjected to flow cytometric analysis for the evaluation of cytotoxicity. Results are the mean±s.d. for three experiments.

**Figure 2:**
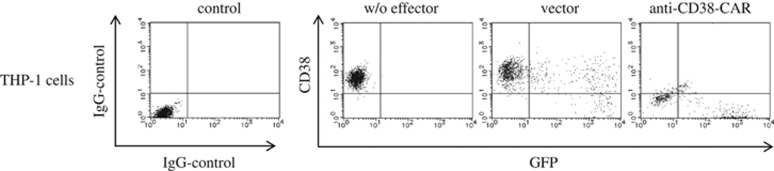
Cytotoxicity of T cells with anti-CD38-CAR against THP-1 cells expressing CD38. Representative results show the cytotoxicity mediated by T cells transduced with the empty vector or the anti-CD38-CAR vector at an E:T ratio of 1:2 for 3 days.

We prepared human peripheral T cells retrovirally transduced with anti-CD38-CAR, as described previously.^13^ First of all, we investigated the expression of green fluorescent protein (GFP) as an internal reference, and an anti-CD38-CAR, which can be cross-reacted with anti-goat mouse IgG-biotin, followed by streptavidin-PerCP on the cell surface of the transduced T cells (data not shown). We confirmed that T cells bearing anti-CD38-CAR expressed GFP as well as PerCP, whose expression is consistent with the expression of anti-CD38-CAR on the transduced T cells (transduction efficiency was 60.90±16.89%, *n*=4). Next, to evaluate the cytotoxic effect of CD38-specific T cells against four AML cell lines, THP-1 and CMK cells highly expressing CD38 (>99% CD38 expression rate), HL60 cells partially expressing CD38 (35.05%) and HEL cells without CD38 expression, the lactate dehydrogenase (LDH) releasing assay was performed. After human T cells transduced with anti-CD38-CAR as an effector (E) were cultured with the respective AML cell line as a target (T) at an E:T ratio of 1:2 for 3 days, the LDH assay showed efficient and specific cell lysis against THP-1 cells and CMK cells by T cells bearing anti-CD38-CAR, but no impact on specific cytotoxicity against HEL cells. Thus, T cells with anti-CD38-CAR eliminated AML cell lines dependent on the expression of CD38 (Table 1 and Figure 1a). Concomitantly, flow cytometric analysis was also performed for evaluation of the cytotoxicity of CD38-specific T cells against THP-1 cells. We co-incubated THP-1 cells with the transduced T cells at a variety of E:T ratios for 3 consecutive days *in vitro*. Interestingly, flow cytometric results showed that most THP-1 cells were abolished by T cells with anti-CD38-CAR at an E:T ratio of 1:2 for 3 days. THP-1 cells were killed in a time- and number of effector-dependent manner (Figures 1b and c), and representative data are shown in Figure 2. However, in the cases of HL60 cells with the partial expression of CD38 and HEL cells without CD38 expression, the LDH assay demonstrated that specific cytotoxicity was 36.96 and 2.22%, respectively (Figure 1a). Results using the LDH assay were well correlated with the expression of CD38 on the cell surface, showing that the cells expressing CD38 were killed by T cells harboring anti-CD38-CAR, and CD38-negative AML cells survived. These results suggest that the augmentation of CD38 is required to induce a higher cytotoxic effect on AML cells not expressing CD38 by T cells with anti-CD38-CAR.

### Enhancement of expression of CD38 and efficacy of the cytotoxicity induced by ATRA

**Figure 3:**
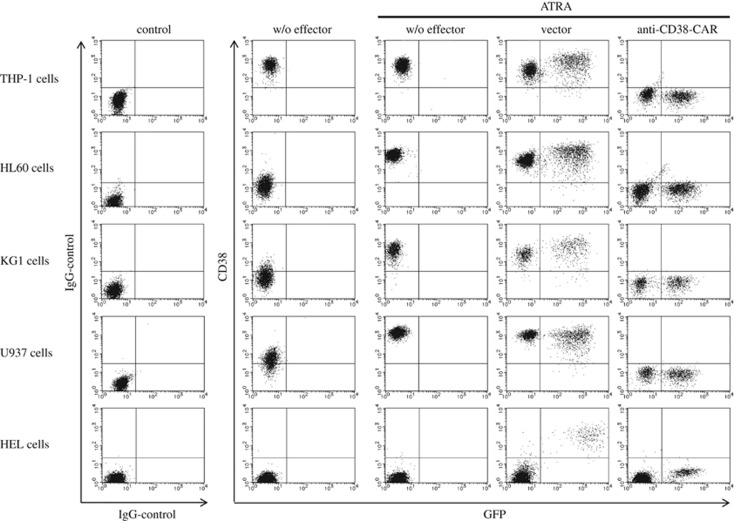
Enhanced expression of CD38 and augmented efficacy of cytotoxicity of anti-CD38-CAR T cells against AML cell line cells by ATRA. AML cell line cells were incubated with or without effectors at an E:T ratio of 1:2 in the presence of ATRA (10 nM) for 3 days. T cells with anti-CD38-CAR killed an AML cell line (far right column), although T cells with an empty vector had no impact on cytotoxicity against the AML cell line, as shown in the fourth column.

**Figure 4:**
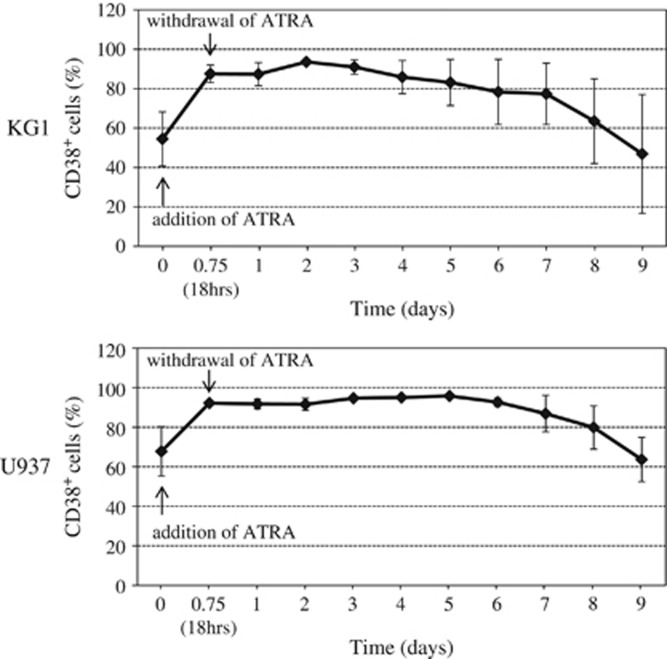
CD38 expression in KG1 and U937 cells after withdrawal of ATRA. These cell lines (KG1 and U937) were cultured in the presence of 10 nM of ATRA for 18 h, and then ATRA was withdrawn from the culture medium. CD38 expression is shown over time. Results are the mean±s.d. for three experiments.

ATRA is widely used for the treatment of APL. In addition, ATRA is also reportedly a potent inducer of CD38 *in vitro*.^17, 18, 19, 20^ We investigated whether ATRA augments CD38 expression and cytotoxicity on several AML cell lines. As shown in Figure 3 and Table 1, even 10 nM of ATRA, whose concentration was much lower than that of serum in patients with the oral administration of ATRA and previously reported *in vitro*,^21, 22^ for 18  h significantly induced the expression of CD38 on the surface of HL60, KG1 and U937 cells. However, the expression of CD38 was not enhanced in HEL cells by ATRA. Moreover, 10 nM of ATRA had no impact on cytotoxicity or proliferating activity in KG1, U937, HEL or even HL60 cells, although viable HL60 cells were significantly decreased in the presence of 1 μM of ATRA for 4 days by Annexin V/PI (propidium iodide) staining (data not shown). In addition, ATRA did not accelerate the harmful effect on these cell lines in a time- or dose-dependent manner in our settings (data not shown). These results show that ATRA contributed to the enhancement of CD38 expression but not to the killing effect on AML cell lines. Moreover, the withdrawal of ATRA from the culture medium decreased CD38 expression in U937 and KG1 cells up to the baseline level before ATRA treatment in ~9 and 13 days, respectively (Figure 4).

**Figure 5:**
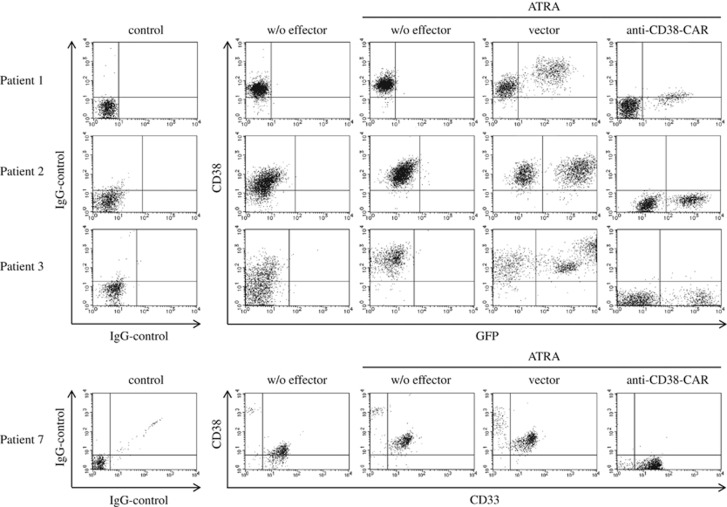
Elevated cytotoxicity of T cells bearing anti-CD38-CAR against AML cells from AML patients dependent on enhancement of CD38 expression by ATRA. AML cells freshly isolated from AML patients (patient 1: M1, patient 2: M4, patient 3: MDS-AML) were cultured with T cells harboring anti-CD38-CAR or empty vector control alone at an E:T ratio of 1:2 in the presence of ATRA for 3 days. T cells with anti-CD38-CAR eradicated AML cells, as shown in the far right column.

Next, we investigated whether CD38 could be enhanced on the surface of AML cells from patients with AML. As shown in Figure 5 (center columns), CD38 expression of AML cells was definitively increased in the presence of ATRA in our settings. These results suggest that CD38 expression was also significantly enhanced by ATRA in AML cells freshly isolated from patients with AML.

### Augmentation of the cytotoxicity by ATRA, followed by CD38-specific T cells against AML cell lines and AML cells from patients

Next, we attempted to estimate the cytotoxicity of CD38-specific T cells against HL60, KG1 or U937 cells following ATRA treatment. AML cell lines were co-cultured with effector T cells for 3 days in the presence of ATRA. As shown in Figure 1a and Table 1, the killing effect of T cells harboring anti-CD38-CAR against AML cell lines was limited without treatment of ATRA, as described above. Intriguingly, T cells with anti-CD38-CAR exerted full cytotoxic effects on AML cells through the enhancement of CD38 expression by ATRA treatment (Figure 3 and Table 1).

Next, we applied our current therapeutic strategy for AML cells freshly isolated from AML patients in our settings described above. As shown in Figure 5 and Table 1, CD38 expression was enhanced by ATRA in AML cells from patients along with AML cell lines, and the 3-day incubation of transduced T cells bearing the anti-CD38-CAR abrogated AML cells from patients' AML cells by the enhancement of CD38 induced by ATRA (Figure 5 and Table 1). These results showed that T cells expressing anti-CD38-CAR efficiently eliminated AML cell lines and AML cells from patients by the enhancement of CD38 expression by ATRA.

## Discussion

We previously developed T cells genetically modified to express an anti-CD38-CAR for CD38-targeted cell therapy. These transduced T cells were cytotoxic to B-lymphoma cells and myeloma cells expressing CD38.^13, 14^ In this study, we demonstrated that T cells transduced with anti-CD38-CAR also showed marked cytotoxicity against AML cells treated with ATRA. This observation is particularly important because it suggests the feasibility of a more effective use of CAR-based immunotherapy combined with the pharmacologic modification of targeted-antigen expression.

For the effective use of T cells transduced with anti-CD38-CAR for AML, the enhancement of CD38 on AML cells is a prerequisite because the expression of CD38 accounts for 83% in AML cells from patients with AML.^10, 11^ Furthermore, the intensity of CD38 expression is much lower than that of B-lymphoma and myeloma cells.^23, 24, 25, 26^ ATRA is clinically available for APL patients through the induction of differentiation and apoptosis in APL cells. Alternatively, it reportedly also promotes the expression of CD38 in APL cells.^17, 18, 19, 20^ Here, we showed that ATRA may be useful for immunotherapy with T cells harboring anti-CD38-CAR through the enhancement of CD38 expression for patients with AML. Indeed, daratumumab, a novel therapeutic human CD38 antibody, and humanized monoclonal CD38 antibody (SAR650984) are currently under clinical use for patients with myeloma without severe side effect.^27^ The concomitant use of an anti-CD38 antibody and ATRA may clinically orchestrate cytotoxicity against AML cells in patients with AML. We demonstrated that robust CD38 expression was induced and enhanced in all sorts of AML cell line by ATRA but not in HEL cells. These cell lines with enhanced CD38 expression were completely abrogated by T cells bearing anti-CD38-CAR, as shown in Figure 3 and Table 1. Intriguingly, a SKY fluorescent *in situ* hybridization assay showed that HEL cells lacked 5p, in which the CD38 gene is located, leading to the absence of CD38 expression on the surface of AML cells even in the presence of ATRA. Next, we investigated whether CD38 expression was induced or enhanced in primary AML cells from the patients by treatment with ATRA. Similarly, CD38 expression was induced and enhanced in AML cells from AML patients in the presence of ATRA. In terms of cytotoxicity against freshly isolated AML cells, T cells with anti-CD38-CAR killed these AML cells from the patients in association with the augmented expression of CD38 by ATRA. Accordingly, we showed that CD38-specific T cells eliminated AML cells through the enhancement of CD38 expression by ATRA. At this point, a question was raised whether HSCs and leukemic stem cells phenotypically expressing CD34^+^CD38^−^ could survive with T cells bearing anti-CD38-CAR in the presence of ATRA. Hence, we need further investigation to clarify the substantial issue on the induction of CD38 with ATRA on the surface of HSCs and leukemic stem cells.

After the co-culture of CD38^+^ AML cells with T cells bearing the anti-CD38-CAR, CD38^−^ AML cells were increased by flow cytometry. Once CD38^+^ cells lost CD38, they were consistent with non-viable cells by PI staining (data not shown). As CD38 was not detected in these cells even using anti-CD38 antibodies, which recognize different epitopes, AML cells losing CD38 were not alive but its mechanism is unclear. However, as we also observed similar results in co-culture of CD19^+^ cells with anti-CD19-CAR T cells,^12^ it seems not to be specific in anti-CD38-CAR T cells. Furthermore, although CD38 was not detected on the surface of anti-CD38-CAR T cells, these are understood as follows: steric change of CD38 by anti-CD38 antibody in the culture medium may be raised on the surface of anti-CD38-CAR T cells.

In summary, we demonstrated that T cells transduced with anti-CD38-CAR effectively eliminated AML cells through CD38 expression induced by ATRA, even though AML cells were negative for CD38. These results may open a new paradigm for pharmacologic inducible immunotherapy that combines ATRA and anti-CD38-CAR for the treatment of patients with AML.

## Methods

### Cells

The AML cell lines THP-1, HL60, KG1 U937 and HEL were purchased from the American Type Culture Collection (Manassas, VA, USA). All cell lines were cultured in RPMI-1640 medium (Sigma, St Louis, MO, USA) supplemented with 10% heat-inactivated fetal calf serum (Gibco-Invitrogen, Carlsbad, CA, USA), L-glutamine, penicillin and streptomycin (Sigma) at 37 ^o^C in 5% CO_2_.

Refractory AML cells (patient 1: FAB M1, patient 2: FAB M4, patient 3: MDS-AML, patient 4: FAB M2, patient 5: FAB M0, patients 6: MDS-AML, patient 7: FAB M5a) were extracted from the bone marrow and peripheral blood after obtaining the appropriate informed consent. CD34^+^ AML cells were isolated from patient 3 with CD34 microbeads kit (Miltenyi Biotec, Bergisch Gladbach, Germany). Patients with AML and two independent donors were examined as approved by the institutional review board at the Hiroshima University. These specimens including more than 90% of AML cells were subjected to Ficoll-Hypaque density centrifugation (GE Healthcare Bio-Sciences, Uppsala, Sweden).

### Vectors, viral production and gene transduction

The retroviral vector containing the GFP as an internal reference, signal peptide, hinge, transmembrane domain of CD8α and intracellular domains of 4-1BB, CD3ζ and anti-CD38 scFv was previously described.^13^ To generate an RD114-pseudotyped retrovirus, we used Lipofectamine-Plus reagents (Invitrogen) to transfect 293 T cells (American Type Culture Collection) with the anti-CD38-CAR-containing vector, pEQ-PAM3(-E), and pRDF (obtained from the St Jude Vector Development and Production Shared Resource, Memphis, TN, USA). Conditioned medium containing the retrovirus was harvested after transfection, and stored at –80 °C until use.

Human peripheral blood mononuclear cells from donors and patients were incubated for 2 h to remove adherent cells. Mononuclear cells were stimulated for 48 h with 7 μg ml^−1^ of PHA-M (Sigma), 200 IU ml^−1^ of human interleukin-2 (PeproTech, London, UK), 10% heat-inactivated fetal calf serum and L-glutamine in RPMI-1640 medium. T cells were suspended in virus-conditioned medium with 4 μg ml^−1^ of polybrene (Sigma) and then centrifuged at 2400 *g* at 32 °C for 4 h in a polypropylene tube coated with retronectin (Takara-Bio, Otsu, Japan). The same procedure was repeated in RPMI-1640 complete medium on the next day. Thereafter, the addition of anti-CD38 antibody was performed to prevent transduced T cells from undergoing autolysis through cross-linking of anti-CD38-CAR with intrinsic CD38.^13^ Briefly, to increase the number of T cells with anti-CD38-CAR, we replaced RPMI-1640 medium containing 10% fetal calf serum, L-glutamine, anti-CD38 antibody (0.15 mg ml^−1^) (CPK-H; MBL, Nagoya, Japan) and interleukin-2 (200 IU ml^−1^) every 4–5 days until 14 days post transduction in order to maintain the viability of the cells. Anti-CD38-antibody was added in the medium for proliferating vector control T cells as well.

During the culture of T cells, transduced T cells were sorted by FACSAria (BD, San Jose, CA, USA) and then cultured again in the presence of anti-CD38 antibody (CPK-H). For the co-culture experiment, T cells were washed with phosphate-buffered saline and complete medium several times to eliminate the effect of residual antibody in the medium.

To detect the surface expression of CAR, cells were stained with a goat anti-mouse (Fab′)_2_ polyclonal antibody conjugated to biotin (Jackson ImmunoResearch Laboratories Inc., West Grove, PA, USA), followed by streptavidin conjugated to peridinin chlorophyll protein (PerCP; BD). The staining was detected with a FACSCalibur flow cytometer (BD), as described previously.^12, 13, 14, 15^

### Cytotoxicity of T cells bearing anti-CD38-CAR *in vitro*

The cytotoxic activity of the transduced T cells was assessed using a flow cytometer, as previously described.^12, 13, 14^ Cells recovered from the culture vessel were stained with an anti-CD38 antibody conjugated to APC (BD). Stained cells were analyzed with a flow cytometer. Using the parental cells, a gating area of forward scatter/side scatter (FSC/SSC) was established. Specific cytotoxicity was evaluated using the formula (B-A)/B, where A is the number of CD38^+^ GFP^−^ cells after incubation with anti-CD38-CAR-expressing T cells, and B is the number of CD38^+^ GFP^−^ cells after incubation with vector-transduced T cells. Instead, the recovery of viable cells (%) was evaluated using the formula A/B.

### Harmful effect of ATRA *in vitro*

To evaluate the harmful effect of ATRA (Sigma), double staining with Annexin V-PerCP (BD) and PI was used. According to the manufacturer's manual, cell pellets (1 × 10^6^ cells) were suspended in 1 × binding buffer. Then, 100 μl of the cell slurry was incubated with Annexin V-PerCP and PI for 15 min at room temperature in the dark. Subsequently, 400 μl of 1 × binding buffer was put into a reaction tube and subjected to FACSCalibur Flow Cytometry.

### LDH cytotoxicity assay

The cytolytic activity of transduced T cells was measured with the LDH-releasing assay using the Cytotoxicity Detection Kit (Takara-Bio). Briefly, leukemic cell lines were incubated with or without T cells transduced with vector alone or anti-CD38-CAR for 18 h at 37 °C in Opti-MEM medium. The supernatant was obtained by centrifugation and then transferred to a microtiter plate. LDH solution was added to the supernatant and then the mixture underwent absorbance measurement. Specific cell lysis was calculated using the following formula: (*A*−*B*−C)/(*D*−*B*−*C*), where *A* is the absorbance of supernatant from an 18-h co-culture of equal amounts of leukemic cell line cells (1 × 10^5^ cells per ml) and the transduced T cells, *B* is the absorbance obtained from the supernatant of leukemic cell line cells alone, *C* is the absorbance obtained from the supernatant of T cells bearing anti-CD38-CAR and *D* is the value obtained from the supernatant of target cells exposed to 1% Triton-X (Sigma). Experiments were carried out in triplicate, and the results were subjected to statistical analysis.

## Figures and Tables

**Table 1:** Cytotoxicity of T cells expressing anti-CD38-CAR against acute myeloid leukemia cells

*Cells*	*CD38 expression (%)*	*CD38 expression following ATRA (%)*	*Specific cytotoxicity ratio (%) following ATRA by FCM*
THP-1	99.99±0.01	99.99±0.01	98.04±0.13
CMK	99.99±0.01	99.99±0.01	96.51±2.72
HL60	35.05±0.61	99.94±0.06	98.92±0.08
U937	75.96±0.47	99.86±0.05	99.80±0.01
KG1	14.21±0.60	99.82±0.09	99.68±0.08
HEL	0.03±0.01	0.08±0.01	2.81±1.72
Patient 1	95.27±0.65	98.00±0.70	99.20±0.01
Patient 2	81.51±0.87	98.72±0.52	99.93±0.01
Patient 3	36.26±0.96	98.09±0.83	99.80±0.03
Patient 4	85.72±0.87	99.22±0.30	99.50±0.17
Patient 5	95.40±1.01	99.38±0.67	99.03±0.30
Patient 6	82.06±0.97	95.40±0.31	93.53±0.67
Patient 7	67.90±2.01	98.31±1.10	99.44±0.11

Abbreviation: FCM, flow cytometry.

Results are the mean±s.d. for three experiments.

Specific cytotoxicity was evaluated by flow cytometry following co-incubation of T cells bearing anti-CD38-CAR (E) with AML cells (T) at an E:T ratio of 0.5:1 for 3 days. We used peripheral T cells from two independent donors for our experiments.
